# The Internet as a Mental Health Advisor in Germany—Results of a National Survey

**DOI:** 10.1371/journal.pone.0079206

**Published:** 2013-11-21

**Authors:** Christiane Eichenberg, Carolin Wolters, Elmar Brähler

**Affiliations:** 1 Department of Psychology, Sigmund Freud University Vienna, Vienna, Austria; 2 Department of Psychology, University of Cologne, Cologne, Germany; 3 Department of Medical Psychology and Medical Sociology, University of Leipzig, Leipzig, Germany; University of Pennsylvania, United States of America

## Abstract

The internet constitutes a popular source of health information. However, the use of the internet and other modern media in the domain of mental health remains widely unclear. This study aimed at exploring the readiness for seeking information online and making use of online counseling and media-assisted psychotherapy. A representative survey of *N* = 2411 Germans was conducted. Results indicated that more than one fourth of Germans would consider seeking help online in case of psychic strain. Participants reported that they would use the internet when needing to research about mental health topics and to communicate with persons concerned on internet forums. Only a small number of participants had already used psychological online-counseling. The majority of subjects reported not having known about the possibility of online counseling. However, the willingness to make use of this option in the future was in a medium range. Concerning the treatment of mental disorders, participants showed a clear preference toward conventional face-to-face treatment. Less than 10% of participants considered the use of treatment supported by mobile phones, the internet, or virtual realities as likely. Certainly, readiness was significantly higher in persons who were already using the relevant devices—mobile phones, computers, and the internet. In the future, there will presumably be an increasing demand for media-assisted psychological counseling and interventions. Members of the health care system should therefore prepare for current developments and help enlighten patients with regard to the possibilities, and also the potential risks of e-mental health.

## Introduction

Seeking health information is one of the most popular purposes of going online [1]. In the USA and UK, the internet constitutes a source of health information for 60–80% of internet users [1], [2] a phenomenon referred to as *e-health*. Data from the Health Information National Trends Survey showed that the internet was the most frequently used source of health and medical information (56%) in the USA, followed by healthcare providers (27%), friends and family (15%), print (12%) and TV (0.6%) [3]. Similarly, more than half (51.1%) of the German population consults the internet for health issues [4]. However, only little is known about the search for information concerning mental health (*e-mental health*). The need for information sources is reflected by the high degree of emotional stress among the German population. A representative study showed that nearly every fourth male and every third female suffered from a mental disorder at the time of the survey [5]. Psycho-educative information from the internet can bridge the long waiting periods for a treatment program in Germany [6] or supplement face-to-face therapy. The internet offers a variety of services, including web-based interventions, online counseling and therapy, internet-operated therapeutic software, and other online activities that can, for example, serve as supplements to face-to-face therapy [7]. The internet is easily accessible and cost effective, and the vast majority of the German population has internet at their disposal [8].

However, a certain proportion of these internet users does not seem to be aware of the options of searching for health information online [9]. Another part of users does not have any demand for health-related information [9]. Certainly, doubts about the quality of online information seem to be a reason for avoiding internet research as well. In fact, the quality of health websites often seems questionable. By way of example, Eichenberg, Blokus, & Malberg [10] evaluated German-speaking websites providing information about posttraumatic stress disorder. They found that information quality was heterogeneous, often due to low usability. Additionally, the impact of online research on health behavior and anxiety should be considered [11]. Then again, a U.S. study showed that not only the internet, but also print media, television, and interpersonal communication generally encourage health-enhancing behaviors [12].

Besides offering a great variety of health-related information, the internet provides the opportunity for *psychological intervention*. Thus persons are able to communicate in self-help groups, to obtain psychological counseling, or to make use of specific intervention programs. Initial empirical studies showed promising results concerning the effectiveness of internet-based psychological interventions in general (e.g. [13], [14], [15], [16], [17]) and online counseling in specific [18], [19], [20] in comparison to conventional face-to-face therapy. Here, too, internet-based interventions represent the advantage of easy and flexible accessibility and wide reach [21]. Moreover, the anonymity of web-based treatment might lower the inhibition threshold of seeking help and enhance openness towards the therapist [22]. A study investigating the acceptability of an internet-based intervention for obsessive-compulsive disorder showed that the most commonly reported advantages were saving time, no need to travel to appointments, reduced cost, and increased privacy. Only few participants reported disadvantages such as preferring face-to-face treatment, perceiving their problems to be too severe for internet-based treatment, not being able to communicating ideas online, and the internet treatment not seeming real [23].

Another approach consists in the use of technological adjuncts to enhance conventional psychotherapy. In so-called *media-assisted psychotherapy* or *m-mental health*, everyday technology such as mobile phones can be utilized to support psychological treatment [24]. For instance, text messages were already used to treat obese elementary school students [25] or to stabilize treatment achievements in patients with bulimia nervosa [26]. Mobile phone-assisted psychotherapy might be a means that is especially suitable for adolescents and young adults, since 96% of Germans between age 12 and 19 possess one [27]. In addition, this kind of treatment may potentially increase therapy compliance [25].

*Virtual realities (VR)* have equally become the focus of attention as an additive of psychotherapy. In behavior therapy, exposure to fear inducing situations can be conducted using VR, which allows a realistic representation of a variety of environments. For example, patients suffering from fear of heights may be confronted with a virtual bridge. VR have proved to be effective in specific phobias, while their use in panic disorder, obsessive-compulsive disorder, and posttraumatic stress disorder await further exploration [28], [29], [30]. In any case, VR provide an opportunity to conduct exposure to anxiety-inducing stimuli at lower financial and logistic costs than conventional in-vivo exposure therapy.

In this sense, there is a broad range of modern media that can be applied in the provision of health information and the treatment of mental disorders. But even though these means have been found to be effective in the treatment of certain mental disorders, the usage and demand of web-based information and interventions offers is still largely unknown. The survey at hand aims at exploring to what extent the German population is already making use of health-related internet offers in general. A further main objective of this study was to reflect the degree of acceptance towards online counseling and media-assisted psychotherapy in case of mental health issues. The findings arising from the survey may help to identify potential population groups who could not have been reached so far. Thus, existing and future information websites and intervention programs can be adapted and developed with regard to interested users. The following research questions were derived from these aims:

1. Which public media are used as health information sources by the German population?
2. How great is the impact of public media on health behavior?
3. To what extent would Germans seek for information and help online in case of emotional distress?
4. To what extent do Germans use psychological online-counseling and how content are persons who have already made use of this service?
5. To what extent do Germans know about the option of getting psychological online-counseling and how great is the willingness to use this service?
6. How great is the willingness to use different kinds of media-assisted psychotherapy?

## Methods

The data were part of the representative study in spring 2010 by USUMA GmbH on behalf of the department of medical psychology and sociology of the University of Leipzig, Germany. The study aimed at exploring psychosocial and social issues that were supposed to be representative for the German speaking residential population of Germany between age 14 and 90 [31]. A representative sample of the German population was selected based on a three stepped drawing of lots, which comprised the selection of sample points, the selection of households, and the selection of persons according to a Kish selection grid. To avoid biases concerning essential sociodemographic characteristics, a population representative loading was carried out. Loading particularly referred to household sizes, diversification of age and gender, and spatial distribution. The survey met the ethical guidelines of the international code of marketing and social research practice by the International Chamber of Commerce and the European Society of Opinion and Marketing Research.

The survey was conducted by 232 trained interviewers in April 2010. Sociodemographic characteristics were inquired as part of the representative survey. The data relevant for this article were gathered using a structured self-report questionnaire within the survey, which had been pretested in a sample of *N* = 67 persons [32].

The survey met the ethical guidelines of the international code of marketing and social research practice by the International Chamber of Commerce and the European Society of Opinion and Marketing Research. The interviewers handed out comprehensive data privacy statements and explicitly pointed out that the survey was completely voluntary and anonymous. All participants gave oral consent. Persons who did not give oral consent were not interviewed, and the number of persons who did not participate was noted down.

### Sociodemographic characteristics

Subjects were questioned about their age and gender, nationality, and affiliation to the east or west part of Germany. Moreover, marital status, living together with a partner, educational level, occupation, household income, and religious denomination were recorded.

### General use of media

The frequency of usage of newspapers, radio, television, telephone, mobile phone, computer or laptop, internet, and minicomputers such as palmtops were rated on a five-point scale from “never” to “daily”.

### Use of health information sources and their impact on health behavior

Participants were asked to state which one of the given sources they would use in order to acquire information and advice about general health issues. In addition, subjects were requested to estimate the impact of the used sources on their behavior on a five point scale from “very low” to “very high”. Dialog partners and media sources included physicians and psychologist, pharmacists, family and friends, the internet, professional literature, health insurances, counseling helplines, and different types of guides in television shows or magazines.

### Use of the internet in case of psychic strain

Participants were asked to report whether the internet would constitute a contact point to obtain information and help in case of psychic strain. If this was answered in the affirmative, they were asked to indicate the type of usage (e.g. research for information about the given health issue, communication in internet forums or looking for contact information of psychotherapists).

### Use of online counseling

Subjects were asked whether they had already made use of psychological counseling on the internet and if not, whether they had known about this possibility. If they had already experienced online counseling, they were requested to indicate their contentment with it on five-point scale from “very discontent” to “very content”. Participants were also asked to report how likely they would believe the use of psychological counseling on the internet in a relevant situation.

### Use of media-assisted psychotherapy in comparison to traditional psychotherapy

The likeliness of the recourse to different forms of therapy was inquired on a five-point scale from “very unlikely” to “very likely”, using the example of a phobia treatment. The following text was presented to the participants.

“Please imagine suffering from an anxiety (phobia), such as fear of heights, spiders, or flying, and being inclined to obtain treatment in order to get rid of your fear. As an adjunct of personal treatment, exercised are sometimes conducted without direct contact to a therapist. At that, media such as mobile phones or computers are often deployed. In the following, four treatment options are presented. Please report if you could imagine getting treated with the aid of each of these options to get rid of your fear.”

1. Conventional psychotherapy
2. Mobile-phone assisted treatment - “i.e. you are trying to overcome your fear by different exercises, e.g. walking over a bridge by yourself if you are suffering from fear of heights. At that you have the possibility to reach your therapist over the phone in case of problems or an emergency. “
3. Treatment using a “virtual reality” - “i.e. the situation that is frightening you is simulated using a computer. Thus you can learn to overcome your fear without having to resort to the actual situation (e.g. the computer simulates a flight situation in case of fear of flying).”
4. Internet assisted treatment – “i.e. you are working through different therapeutic exercises, being supported by a therapist via the internet.“

Statistical analysis of the survey was realized using the computer program SPSS Statistics version 19.0. The survey data were analyzed using descriptive as well as inferential statistical methods. Relationships between categorical data were analyzed using Chi-squared test. Associations between interval-scaled data were analyzed by means of Pearson Product Moment Correlation.

## Results

*N* = 2411 subjects of German citizenship took part in the survey. Participants had an average age of *M* = 51 years (*SD* = 18.6); 53.2% (*n* = 1298) of them were female and 46.8% (*n* = 1113) were male. These numbers are consistent with those of a study by a renowned market research institute which found that 52.9% of the German population is male and 47.2% is female [33], proving the representativeness of our sample.

Furthermore, roughly half of the subjects (53.2%, *n* = 1128) stated being married, while 46.8% (*n* = 1128) declared being single, divorced, or widowed. Moreover, just under half of the participants (47.7%, *n* = 1150) worked full-time or part-time.

### General use of public media

The large majority of Germans indicated using conventional public media such as newspaper (59.5%, *n* = 1430), radio (66.4%, *n* = 1594), television (81.3%, *n* = 1953) and telephone (53.5%, *n* = 1283) daily. Among the remaining participants, the bigger part reported using the relevant public media several times a week. A minority of persons never uses any of the given public media. For instance, merely 1.5% (*n* = 36) stated they never watch television. In contrast, while 31.7% (*n* = 7619) of Germans use a computer or laptop daily, 41% (*n* = 983) never use any (see table 1). Moreover, results indicated that mobile phones and the internet have become everyday public media: 75.8% (*n* = 1822) claimed to use a mobile phone and 58.8% (*n* = 1413) the internet (see table 1). On the contrary, minicomputers are used by merely 8% (*n* = 205) altogether.

**Table 1:** Frequency of usage of different media in % *(n)*.

	Daily	Several times a week	A few times a month	Less frequently	Never
Computer/Laptop	31.7 (761)	18.4 (441)	5 (121)	3.9 (94)	41 (983)
Internet	28.7 (690)	19.5 (469)	6.3 (151)	4.3 (103)	41.2 (991)
Mobile phone	35.4 (852)	22.5 (541)	10.7 (258)	7.1 (171)	24.2 (583)

1. Which public media are used as health information sources by the German population?

**Figure 1:**
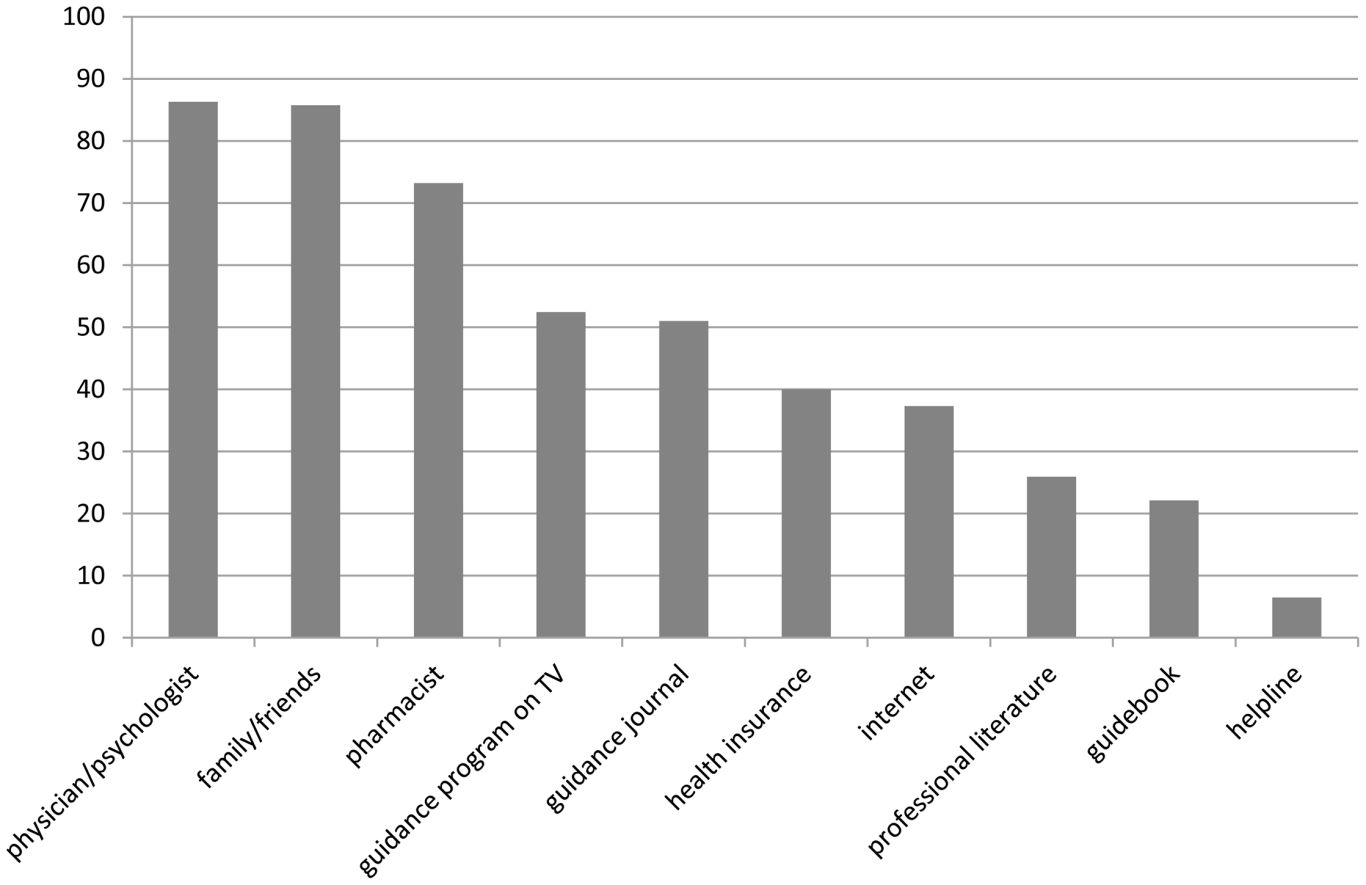
Portion of users of different health information sources (in %, N = 2411).

The majority of Germans consults traditional contact persons such as physicians, psychologists, pharmacists, as well as family and friends, to get advice or information about general health issues. The internet is used for health related questions by 37.7% (*n* = 897) of the German population (see figure 1). Among those who use the internet at all, almost two thirds (64.5%, *n* = 895) use the internet for health information. Thus the internet takes on a more important role in providing health information than guides on television, in journals, or in books.

2. How great is the impact of public media on health behavior?

Participants rated the impact of frequently used sources on health behavior as higher than that of seldom used ones. As table 2 shows, the impact of physicians, psychologists, family members, and friends on health behavior is considered relatively high by the majority of Germans. In contrast, the internet seems to have a mediocre impact on health related behavior, comparable to that of health insurances and guidebooks. However, about one fifth of those who use the internet as a source of health information consider its impact on health behavior as “high” (13.2%, *n* = 117) or “very high” (5.2%, *n* = 46).

**Table 2:** The impact of different information sources on health behavior in Germany.

Source	*M (SD)* *	*n* **
Physicians/psychologists	3.9 (0.9)	2064
Family/friends	3.3 (1.0)	2047
Pharmacists	3.0 (1.0)	1744
Professional literature	3.0 (1.1)	618
Internet	2.7 (1.0)	889
Health insurances	2.7 (1.0)	948
Guidebooks	2.7 (1.0)	525
Guidance journals	2.5 (0.9)	1211
Helplines	2.4 (1.0)	150

* 1: very low impact; 5: very high impact.

** Number of participants who reported to use the relevant source at all.

3. To what extent would Germans seek information and help online in case of emotional distress?

More than one fourth of Germans (26.3%, *n* = 635) could imagine seeking help online in case of emotional distress, corresponding to 43.7% of internet users. The large majority of those (90.3%, *n* = 558) indicated researching on mental health topics in case of need. Other reasons for using the internet in case of emotional distress were the communication with other persons concerned on internet forums (40.8%, *n* = 252), searching for psychological online tests (28.2%, *n* = 174), and acquiring contact data of resident psychotherapists (30.6%, *n* = 189). Several socio-demographic factors determined the probability of taking the internet into account in case of emotional distress. Young (χ^2^ = 3.29, *df* = 6, *p*<.001) and single (χ^2^ = 1.54, *df* = 4, *p*<.001) persons with a final degree (χ^2^ = 63.78, *df* = 1, *p*<.001) and an income above 2500 (χ^2^ = 96.19, *df* = 3, *p*<.001) were most likely to come back to the internet in case of need (all *p*<.001). Additionally, persons using the internet very frequently were more likely to utilize it in case of emotional need than persons using it only several times a month (χ^2^ = 5.92, *df* = 4, *p*<.001).

4. To what extent do Germans use psychological online-counseling and how content are persons who have already made use of this service?

Merely 2.2% (*n* = 14) reported already having used psychological online counseling. Contentment with this form of counseling was indicated to be fairly good, with an average of *M* = 3.9 (*SD* = 0.7) on a scale from 1 (“very discontent”) to 5 (“very content”).

5. To what extent do Germans know about the option of getting psychological online-counseling and how great is the willingness to use this service?

Scarcely half (43.7%, *n* = 274) of those who would use the internet in case of emotional distress stated knowing about the option of online counseling. Therefore, the majority of persons who are open to psychosocial support on the internet (54.1%, *n* = 339) initially learned about the possibility of online counseling by the survey. The likelihood of a future use of this services was reported to be *M* = 2.8 (*SD* = 1.0) on a scale from 1 (“very unlikely”) to 5 (“very likely”), with the largest portion (42.0%, n = 264) answering “maybe”.

Only few socio-demographic factors distinguished those who would know about the option of online counseling from those who did not: Persons with a final degree (χ^2^ = 6.06, *df* = 1, *p*<.05), persons in rural areas (χ^2^ = 4.42, *df* = 1, *p*<.05) and persons with a religious affiliation (χ^2^ = 10.48, *df* = 1, *p*<.01) were more likely to know about the possibility of getting psychological counseling on the internet.

6. How great is the willingness to use different kinds of media-assisted psychotherapy in comparison to traditional psychotherapy?

In relation to the question which kind of treatment they would use in case of an actual mental disorder, participants showed a clear preference towards conventional personal treatment. More than half of the German population (52.7%; *n* = 1264) considers its use in case of need “rather” or “very likely”. In contrast, merely 7.6% (*n* = 183) thought the use of mobile phone assisted treatment to be “rather” or “very likely”. Similar results were found concerning internet assisted psychotherapy and treatment using a virtual reality (see table 3). However, readiness was significantly higher in persons who were already using the relevant devices- mobile phone, internet, or computer. In addition, there were significant correlations between the willingness to use treatment supported my mobile phones, virtual reality, or the internet, and the frequency of use of the relevant media: mobile phones (*τ_b_* = .21, *p*<.001, *n* = 2389), computer (*τ_b_* = .26, *p*<.001, *n* = 2382), and internet (*τ_b_* = .32, *p*<.001, *n* = 2387).

**Table 3:** Willingness to use media assisted psychotherapy on a scale from 1 (“very unlikely”) to 5 (“very likely”).

Treatment options	*M (SD)* * *N* = 2411	*M (SD)* *, *n* **	t-Test: *t*, *df*, *p*, *n*
Conventional personal treatment by a therapist	3.4 (1.3)		
Mobile phone assisted treatment	1.8 (1.0)	1.9 (1.1), *n* = 1816	*t* = −10.23, *df* = 1193.94, *p*<.001, *n* = 2389
Treatment by means of a “virtual reality”	1.7 (1.0)	2.0 (1.1), *n* = 1412	*t* = −15.38, *df* = 2378.86, *p*<.001, *n* = 2389
Internet assisted treatment	1.7 (1.0)	2.0 (1.1), *n* = 1408	*t* = −19.61, *df* = 2303.53, *p*<.001, *n* = 2387

* 1: very unlikely; 5: very likely.

** Only users of the relevant media (mobile phone, computer, internet) were included.

Persons with high readiness (“rather” or “very likely” use) and low readiness (“rather” or “very unlikely” use) to use conventional psychotherapy or media assisted treatment differed in several socio-demographic factors (see table 4).

**Table 4:** Association between the willingness to use different kinds of media assisted psychotherapy and socio-demographic factors.

Variable		?^2^- Test: χ^2^, *df*, *p*	*n*	Significance
Sex	Conventional	?^2^ = 17.91, *df* = 2, *p*<.001	2411	***
	Mobile phone assisted	?^2^ = 7.14, *df* = 4, *p* = .13	2395	n.s.
	Virtual reality	?^2^ = 5.93, *df* = 4, *p* = .20	2393	n.s.
	Internet assisted	?^2^ = 8.17, *df* = 4, *p* = .09	2393	n.s.
Age	Conventional	?^2^ = 64.88, *df* = 12, *p*<.001	2411	***
	Mobile phone assisted	?^2^ = 1.90, *df* = 24, *p*<.001	2395	***
	Virtual reality	?^2^ = 2.34, *df* = 24, *p*<.001	2393	***
	Internet assisted	?^2^ = 2.78, *df* = 24, *p*<.001	2393	***
Family status	Conventional	?^2^ = 23.69, *df* = 8, *p*<.01	2411	**
	Mobile phone assisted	?^2^ = 89.44, *df* = 16, *p*<.001	2395	***
	Virtual reality	?^2^ = 1.23, *df* = 16, *p*<.001	2393	***
	Internet assisted	?^2^ = 1.47, *df* = 16, *p*<.001	2393	***
Educational level	Conventional	?^2^ = 5.07, *df* = 2, *p* = .079	2411	n.s.
	Mobile phone assisted	?^2^ = 5.36, *df* = 4, *p* = .25	2395	n.s.
	Virtual reality	?^2^ = 9.51, *df* = 4, *p* = .05	2393	*
	Internet assisted	?^2^ = 19.82, *df* = 4, *p*<.01	2393	**
Income	Conventional	?^2^ = 13.29, *df* = 4, *p*<.05	2328	*
	Mobile phone assisted	?^2^ = 35.92, *df* = 12, *p*<.001	2315	***
	Virtual reality	?^2^ = 61.45, *df* = 12, *p*<.001	2313	***
	Internet assisted	?^2^ = 71.83, *df* = 12, *p*<.001	2393	***
City/Rural area	Conventional	?^2^ = 81, *df* = 2, *p* = .669	2411	n.s.
	Mobile phone assisted	?^2^ = 26.76, *df* = 4, *p*<.001	2395	***
	Virtual reality	?^2^ = 14.10, *df* = 4, *p*<.01	2393	**
	Internet assisted	?^2^ = 2.86, *df* = 4, *p* = .58	2393	n.s.
Religion	Conventional	?^2^ = 12.94, *df* = 2, *p*<.01	2401	**
	Mobile phone assisted	?^2^ = 35.92, *df* = 4, *p* = .28	2385	n.s.
	Virtual reality	?^2^ = 5.65, *df* = 4, *p* = .23	2393	n.s.
	Internet assisted	?^2^ = 2.26, *df* = 4, *p* = .69	2393	n.s.

n.s.: not significant.

* p<0.1;

** p<0.01;

*** p<0.001.

While significantly more women (56.3%, *n* = 731) than men (47.9%, *n* = 553) stated willingness to use conventional psychotherapy, there were no gender differences regarding the use of media assisted treatment. There were significant differences concerning the *age* of the respondents. Persons in the age of 25 to 34 were most likely to make use of conventional therapy in case of need (“rather” or “very likely” use: 59.1%, *n* = 175). Readiness to use conventional face-to-face therapy was smaller in older persons, with less than half of persons (48.2%, n = 201) in the age group of 65 to 74 reporting to be willing to use conventional psychotherapy. The portion of persons under the age of 25 who would consider using conventional psychotherapy is similar to that (46.3%, *n* = 105). In contrast, young adults were more likely to use media-assisted treatment than all of the other age groups. For instance, while 11.5% (*n* = 26) of persons under 25 years consider the use of mobile phone-assisted treatment “rather” or “very likely”, merely 7.9% (*n* = 34) of 45- to 54-year-olds do so.

*Family status* was another factor differentiating between high and low readiness to use different forms of therapy. For instance, person who are married and living together (54.7%, *n* = 691) or divorced (55%, *n* = 127) considered conventional treatment more likely than single persons (51.5%, *n* = 290). At the same time, singles were more willing to make use of media-assisted psychotherapy.

With respect to *educational level*, there were noticeable differences in the willingness to use internet-assisted psychotherapy. Persons with a final degree seem to consider it more likely to use this form of treatment (7.4%, *n* = 25) than persons without a degree (5.5%, *n* = 113). Similarly, Germans with an income of more than 2500 Euro per month reported having a higher readiness to use all kinds of media-assisted treatment in comparison to those with a lower income.

Furthermore, persons living in the *city* regarded the use of. mobile phone-assisted treatment, for example, significantly more often as “rather” or “very unlikely” (76.3%, *n* = 1587) than those living in *rural areas* (68.6%, *n* = 216). Finally, persons who *belong to a church* stated considering the use of conventional therapy more likely than those who do not.

Results also showed significant associations between the use of media-assisted therapy and the *use of different sources to obtain health information*. For instance, there is a significant association between the use of the internet for health-related questions, and the willingness to use all of the three forms of media-assisted therapy. Persons who are using the internet to research for health information reported more often considering the use of a mobile phone-assisted treatment “rather” or “very likely” (11.9%, *n* = 106) than persons who are not using the internet for this purpose (5%, *n* = 75) (χ^2^ [4, *n* = 2387] = 130.42, *p*<.001). There was a similar distribution with regard to internet-assisted treatment and treatment by means of a virtual reality.

The perceived impact of different information sources on health behavior seems to be associated with the readiness to use media-supported psychotherapy as well. For example, persons who considered the impact of the internet on their health behavior as high estimated the use of a virtual reality treatment more often as “rather” or “very likely” (24.8%, *n* = 29) than those who rated the impact of the internet as low (8.6%, *n* = 24) (χ^2^ [8, *n* = 889] = 55.40, *p*<.001).

## Discussion

In the past years, modern technologies have gained in importance in the field of psychotherapy. The potential benefits of different technological adjuncts of therapy such as the internet, virtual realities, or mobile phones could be proved in a variety of studies.

However, to date, acceptance of and readiness to use media-assisted psychotherapy in the general population has been unresolved.

The study at hand aimed at filling this gap of knowledge by conducting the first population representative survey exploring the use of modern media concerning health information, psychological counseling, and therapy. Results indicated that the internet has become an important source of health information. Almost two thirds of internet-users resort to it for health-related questions. Admittedly, the self-reported impact of the internet on concrete health behavior is low in comparison to traditional dialog partners such as physicians, psychologists, and family members.

Findings also showed a relatively high readiness to use the internet in case of mental health problems. One fourth of the general population and almost one half of internet-users would use e-mental health offers in case of need. Favored offers include online health websites and online self-help groups. Particularly young, well-educated single persons seem to consider the use of such offers as likely. These results are in accordance with general findings concerning the association between socio-demographic variables and the willingness to use any kind of psychological support (for a U.S. study, see [34]; for Germany, see [35], [36]). However, while the majority of studies exploring the current state of psychological care show a superior number of women, the present study indicates a balanced gender ratio with regard to the use of the internet in case of mental health problems. At the same time, the largest portion of Germans seems not aware of the opportunities that the internet provides in the field of mental health care. More than half of persons who are open towards psychological support in the internet did not know about the offer of online counseling. However, there is a general willingness to use psychological online counseling in the future.

In contrast, media-supported psychotherapy does not seem too well accepted with mental disorders in need of treatment. Conventional psychotherapy is preferred by the vast majority of Germans. Despite positive evaluations of treatments with the aid of virtual realities as well as internet-based treatment programs, face-to-face communication with a therapist seems to be indispensable in their view. Admittedly, the willingness to use technological adjuncts of therapy increases, if the relevant device is already used. As a consequence, the advancing use of modern technologies may be accompanied by an increasing demand of e-mental health. Therefore, health politics, research, and practice should be prepared for corresponding developments. The population should be enlightened not only with respect to the possibilities and advantages, but also concerning the potential risks and disadvantages of e-mental health. For instance, online mental health information and self-tests may lead to false diagnoses or to self-stigmatization of the persons concerned. In addition, if websites are not supervised by health professionals, information may lead to the wrong intervention or to no intervention at all. Mental health information on the internet is indeed easily accessible and cost effective, but not necessarily helpful. Therefore, it is particularly important that clinical research and practice figure out for which patients and in which state of disorder e-mental health can be useful. The present study helped close that gap of knowledge by identifying socio-demographic characteristics associated with the use and the willingness to use online health sources.

Concomitant with the growing number of possible ways of accessing mental health information, it is getting evermore difficult to estimate the quality of respective offers. The development and establishment of quality seals for mental health websites thus seems essential. Another important research topic is the identification of variables that enhance the adherence to internet-based intervention in order to minimize dropout rates [37].

Moreover, physicians and psychologists should know how to react to internet-informed patients. In the future, patients might be increasingly more informed about their disorders, make use of internet offers such as online counseling, or voice their intention to involve modern technologies in the treatment. Health care providers should thus be open towards changes in the help system. Ideally, public media and technologies should be included in the treatment, whilst taking into account general empirical evidences as well as individual patient characteristics.

Finally, a few potential limitations of the present study should be addressed. Primarily, a study by the Federal Statistical Office [8] found different numbers concerning the participants' use of computers (16% in comparison to 41% in our study) and the internet (21% in comparison to 41.2%). However, we referred to the present internet use, while they asked whether the internet had ever been used before. In addition, the Federal Statistical Office did not report the response rate of their study, which might have been lower than in our study.

Secondly, our study was based on self-report data. Even though there were no indications for this, participant responses might in some cases be biased by effects of memory or social desirableness. Therefore, future studies could include objective data such as the actual frequency of consultations when examining the impact of public media on health behavior. However, the present study aimed at examining the willingness to use different e-mental health offers, a question that can only be assessed by self-report data. Another difficulty in survey studies with large samples is the need to keep the inquiry rather compact. Therefore, it was not possible to address some of the subjects in depth. More extensive studies, particularly qualitative survey studies, might, for example, explore the reasons for the willingness or unwillingness to use certain media. In addition, the cross-sectional design of the present study does not allow drawing conclusions about the course of media use with advancing age, but only comparing their use across different age groups. Finally, the question that was asked to explore the willingness to use media-assisted therapy specifically referred to the treatment of anxiety. The answers to this question might thus not be transferrable to the whole spectrum of mental disorders. However, anxiety represents a typical application area for media-supported psychotherapy and was therefore chosen as an example. In addition, the descriptions of the different forms of media-assisted therapy included typical psychotherapeutic methods used in traditional face-to-face therapy. Therefore, if participants reported unwillingness to use one type of method, it is unclear whether they are not willing to use the specific component or psychotherapeutic methods on the whole. Certainly, the proportion of participants who reported being willing to engage in traditional face-to-face therapy was much higher than that of persons who indicated willingness to use media-assisted psychotherapy. It could thus be assumed that in most participants, the unwillingness to use media-supported methods can be ascribed to the specific media components, and not to a general rejection of the psychotherapy.
